# Researchers’ Ethical Concerns About Using Adaptive Deep Brain Stimulation for Enhancement

**DOI:** 10.3389/fnhum.2022.813922

**Published:** 2022-04-14

**Authors:** Kristin Kostick-Quenet, Lavina Kalwani, Barbara Koenig, Laura Torgerson, Clarissa Sanchez, Katrina Munoz, Rebecca L. Hsu, Demetrio Sierra-Mercado, Jill Oliver Robinson, Simon Outram, Stacey Pereira, Amy McGuire, Peter Zuk, Gabriel Lazaro-Munoz

**Affiliations:** ^1^Center for Medical Ethics and Health Policy, Baylor College of Medicine, Houston, TX, United States; ^2^Center for Medical Ethics and Health Policy, Baylor College of Medicine, Rice University, Houston, TX, United States; ^3^Anthropology & Bioethics Department of Social & Behavioral Sciences, Institute for Health & Aging, University of California, San Francisco, San Francisco, CA, United States; ^4^Department of Anatomy & Neurobiology School of Medicine, University of Puerto Rico, San Juan, Puerto Rico; ^5^School of Medicine, University of California, San Francisco, San Francisco, CA, United States; ^6^Center for Bioethics, Harvard Medical School, Boston, MA, United States; ^7^Department of Psychiatry, Massachusetts General Hospital, Boston, MA, United States

**Keywords:** neuroenhancement (NE), neurotechnology and brain-machine-interface, adaptive deep brain stimulation, perspectives, qualitative study

## Abstract

The capacity of next-generation closed-loop or adaptive deep brain stimulation devices (aDBS) to read (measure neural activity) and write (stimulate brain regions or circuits) shows great potential to effectively manage movement, seizure, and psychiatric disorders, and also raises the possibility of using aDBS to electively (non-therapeutically) modulate mood, cognition, and prosociality. What separates aDBS from most neurotechnologies (e.g. transcranial stimulation) currently used for enhancement is that aDBS remains an invasive, surgically-implanted technology with a risk-benefit ratio significantly different when applied to diseased versus non-diseased individuals. Despite a large discourse about the ethics of enhancement, no empirical studies yet examine perspectives on enhancement from within the aDBS research community. We interviewed 23 aDBS researchers about their attitudes toward expanding aDBS use for enhancement. A thematic content analysis revealed that researchers share ethical concerns related to (1) safety and security; (2) enhancement as unnecessary, unnatural or aberrant; and (3) fairness, equality, and distributive justice. Most (70%) researchers felt that enhancement applications for DBS will eventually be technically feasible and that attempts to develop such applications for DBS are already happening (particularly for military purposes). However, researchers unanimously (100%) felt that DBS ideally should not be considered for enhancement until researchers better understand brain target localization and functioning. While many researchers acknowledged controversies highlighted by scholars and ethicists, such as potential impacts on personhood, authenticity, autonomy and privacy, their ethical concerns reflect considerations of both gravity and perceived near-term likelihood.

## Introduction

The capacity of next-generation deep brain stimulation (DBS) devices such as closed-loop or adaptive DBS (aDBS) to both read (measure neural activity) and write (stimulate a brain region or circuit) in response to measured neural activity could improve the management of movement (e.g., Parkinson’s disease, essential tremor, dystonia), seizure, and psychiatric disorders (e.g., obsessive compulsive disorder, Tourette). However, the capacity to read and write also opens the door to attempts to electively modulate mood, cognition, and prosociality for non-therapeutic reasons. Considered within a context of increasing cultural acceptance and optimism toward the use of technology for self-improvement (Jasanoff, 2015) and growing direct-to-consumer marketing of neurotechnologies for enhancement purposes (Kreitmair, 2019; Wexler and Reiner, 2019), it is unsurprising that the positive outcomes associated with aDBS have piqued interest in its potential to be expanded beyond treatment applications into the realm of enhancement in non-clinical populations. While a range of other non-invasive neurotechnologies (e.g., electroencephalography, transcranial electrical stimulation, etc.) are likewise being explored for use as enhancements (Kreitmair, 2019; Wexler and Reiner, 2019), we focus here specifically on aDBS, which differs from most other neurotechnologies by virtue of being an invasive, surgically implanted technology with an established capacity to both read and write to the brain, meaning the device can monitor as well as independently and automatically stimulate brain activity. This closed-loop technology, once further developed, would bypass the need for active monitoring or intervention by a human agent and raise ethical questions related to data privacy and security, patient informed consent and understanding (Muñoz et al., 2020), and the need to preserve personal autonomy (Zuk and Lázaro-Muñoz, 2019a), agency, and identity (Lázaro-Muñoz et al., 2017). When used in the context of experimental clinical settings, aDBS devices raise additional concerns about post-trial continued access (Muñoz et al., 2020) to and/or removal of devices (Sierra-Mercado et al., 2019) for participants in research trials after the study period is over. The intentional expansion of aDBS technology into the commercial sphere for non-clinical and elective (Neuralink Inc, 2022) uses evokes further concerns, both practical (related to function and efficacy) and ethical (related to moral rightness or wrongness), revolving around safety (that it could potentially harm consumers) (Jarchum, 2019), data security/privacy (brain activity could be accessed by unwanted third parties, “hacked” or otherwise exploited or exchanged as “currency” via licensing agreements) (Dresler et al., 2018), and free will (that neuroenhancement may threaten our understanding of human agency, responsibility and liability) (Nahmias et al., 2014; Klein et al., 2016).

There is disagreement over the urgency of addressing ethical concerns related to the use of aDBS for enhancement, with some arguing that the prospect is “just around the corner” (Farah et al., 2004) and others arguing it may never happen or will take decades (Schlaepfer and Fins, 2010; Wexler, 2019). Part of this disagreement stems from a lack of clarity about what aDBS systems are (or will be) capable of. For example the Neuralink corporation is developing a system to allow users to communicate with and even operate electronics and/or robotics with their mind (Musk, 2019). While developers plan for a “first wave” of treatment-only applications intended to assist functioning in individuals with limited motor function or difficulty in communicating, its features are expected to also appeal widely to non-disordered or “normally” functioning individuals (Dadia and Greenbaum, 2019; Steinert et al., 2019). Neuralink is projected to begin first-in-human trials this year (Novet, 2019). Other DBS technologies are explicitly being developed for non-clinical use, including Imec’s Neuropixels (Jun et al., 2017) technology for recording neural activity and transmitting digital data outputs to the brain, as well as a wide range of brain-machine interface (BMI) initiatives funded by the Defense Advanced Research Projects Agency [DARPA] (2020) to enable, for example, data transfer between the brain and digital applications, accelerate and improve functional behaviors, advance the speed and effectiveness of learning cognitive skills through targeted stimulation, and facilitate memory formation and retrieval.

Certainly, many of the concerns surrounding these emerging aDBS and related neurotechnologies may be fully warranted as we survey their rapid pace of development. However, the extent to which we should focus efforts at mitigating particular risks over others in an anticipatory fashion should depend not only on the potential gravity of their impacts but also on their differential probabilities of occurring. This central calculus of risk management (Manuele, 2016) implores us to get an “insider view” on research and development of these technologies. For this critical reason, we focus on reporting perspectives and attitudes toward the use of aDBS for enhancement from among researchers who are actively researching, developing and observing aDBS technology’s specific impacts on human subjects involved in research trials. These key stakeholders are uniquely positioned to provide important insights into the opportunities and limitations of aDBS systems and to help prioritize the potential ethical and practical concerns they may raise (Muñoz et al., 2020). Here, we explore which concerns they feel are most legitimately and urgently raised by the use of aDBS for enhancement purposes. To our knowledge, this paper represents the first empirical examination of this question.

## Materials and Methods

### Participants

We conducted semi-structured, open-ended interviews via phone or Zoom with researchers (*n* = 23; out of total of 28 contacted, for a response rate of 82%) involved in aDBS trials across six different research institutions in the United States. These individuals were recruited from funded aDBS trials and possess expert knowledge about these devices, have direct experience developing and/or implementing them and observing their effects among individuals from a variety of clinical populations, including those suffering from Parkinson’s disease, dystonia, essential tremor, Tourette syndrome, obsessive-compulsive disorder, and depression. In order to ensure recruitment of different project roles of researchers (including senior researchers) involved in aDBS trials (e.g., trial coordinators, neurologists, neurosurgeons, psychiatrists, and engineers) (See Table 1), we employed purposeful snowball sampling (Palinkas et al., 2015), whereby we asked interviewees to refer to us any colleagues with relevant expertise. The study was approved by the Institutional Review Board at Baylor College of Medicine.

**TABLE 1:** Researcher demographics.

Gender (*n* = 23)	
Male	13 (57%)
Female	9 (39%)
Prefer not to answer	1 (4%)
**Race/Ethnicity (*n* = 23)**	
Asian	3 (13%)
White	18 (78%)
Prefer not to answer	2 (9%)
**What degree(s) do you currently hold? (*n* = 23)**	
M.D. or equivalent	8 (35%)
Ph.D. or equivalent (clinical)	3 (13%)
Ph.D. or equivalent (research)	4 (17%)
Both M.D. and Ph.D. or equivalent (clinical)	2 (9%)
Both M.D. and Ph.D. or equivalent (research)	1 (4%)
B.Eng. or M.Sc. Engineering	2 (9%)
B.A. or B.S.	3 (13%)
**Project roles (*n* = 23)**	
Clinical trial coordinator	4 (17%)
Engineer	5 (22%)
Mental health clinician	4 (17%)
Neurologist	5 (22%)
Neurosurgeon	5 (22%)
**Research focus (*n* = 23)**	
Movement disorders	6 (26%)
Psychiatric disorders	8 (35%)
Both	9 (39%)
**Mean years of research experience (*n* = 23)**	
Years of experience related to conventional DBS	8.7
Years of experience related aDBS	4.5

### Interview Constructs

We asked respondents to share their perspectives in response to two questions: (1) Do you think adaptive DBS could be used not only for the purpose of treatment, but also for enhancement? (to gauge the perceived likelihood and imminence of using aDBS for enhancement); and (2) How do you feel about potentially using these technologies for enhancement? (to identify any practical, ethical or other concerns raised by the use of aDBS for enhancement). Based on Parens’s (Parens, 1998) definition, we defined enhancement for our respondents as any technological or biomedical intervention used to improve someone’s cognitive, motor, or moral abilities beyond those of the average person, and beyond what is necessary to restore or sustain health. These questions were part of a larger interview guide developed to explore key ethical issues surrounding aDBS research, which we report elsewhere (Muñoz et al., 2020; Zuk et al., 2020).

### Analysis

Interviews lasted an average of 56 min and were audio-recorded, transcribed verbatim, and analyzed with the aid of MAXQDA 2018 software (Kuckartz and Rädiker, 2019). Each interview transcript was coded independently by at least two members of the research team. Inconsistencies in coding were discussed to reach consensus among the research team. Utilizing thematic content analysis (Boyatzis, 1998; Schilling, 2006), information from coded segments was progressively abstracted to identify the content and frequency of emergent themes, with higher frequency themes reported here as primary ethical concerns. Frequencies for expressed attitudes account for overlap between attitudinal categories (e.g., positive vs. negative vs. neutral/ambivalent) in two ways: Where a respondent expressed more than one perspective, we revisited the relevant quotations in order to interpret which stance a respondent seemed to lean most toward. In some cases, this second analysis of the quotations allowed for a resolution about which attitudinal category to assign, while in other cases we felt that the strength of a respondent’s ambiguity warranted characterizing that respondent’s view as ambivalent (i.e., “maybe” in Table 2) or “overlapping” (Table 3).

**TABLE 2:** aDBS researchers’ perspectives on whether aDBS could be used for enhancement.

Yes	16	70%
No	5	22%
Maybe	2	9%
TOTAL	23	100%

**TABLE 3:** aDBS researchers’ primary ethical concerns about using aDBS for enhancement.

Total *n* = 23 aDBS researchers	
**Safety and security concerns (61%)**	
Inherent risks and invasiveness	“I don’t think it would have wide uptake. I don’t think there would be a great deal of interest in using DBS for enhancement using the current types of technology, which require implanting electrodes in the substance of the brain. It would have to be less invasive.”
Inherent risks and invasiveness	“It is still somewhat of an invasive procedure, so hopefully that will be somewhat of a deterrent for people that are otherwise healthy and functioning at a high level.”
Inherent risks and invasiveness	“These are implanted devices [with] inherent risk. It’s not minimal if some adverse consequence were to happen. The ethical concern would be that it was not fully vetted to the extent that we felt like it was a safe procedure to do. I would have questions for mass implantation of people to make them smarter or happier or both.”
Inherent risks and invasiveness	Given these risks, most researchers echo one researcher’s comment that “I don’t think it’s really appropriate to use an invasive therapy for enhancement personally. There’s so many risks involved.”
Risk/benefit ratio	“I think we’d find it unethical to do so in the absence of disease or any indication to have brain surgery for it.”
Risk/benefit ratio	Even though [DBS surgery] is considered quite safe, it’s not devoid of potential risk. There is risk of brain bleeding, risk of stroke, risk of brain infection. And so, one has to have a very good reason to undergo such treatment, such as having a disease that is well studied, that with effective treatment there is a good chance that the symptoms are going to improve, or the disease can be modified in some way.
**Enhancement as unnecessary, unnatural or aberrant (43%)**	
Fundamental changes: person	“There’s definitely a potential that it could be used in that way [enhancement]… as a cognitive enhancer, for example. It’s a little bit scary to think about that. At what point do we go from being people to, I don’t know, augmented humans?”
Fundamental changes: society	All of this work we’re doing on depression and anxiety is enhancement in a way. It’s enhancement of mood, and we think that’s a reasonable thing to do because we’re treating people’s symptoms. But is it better if everybody’s a little happier? Do you just want everybody to be happy all the time, then we’re changing society at that level too. It’s a broader question.
**Fairness, equality and distributive justice concerns (35%)**	
Socioeconomic advantages	“If it really could change how people are successful at life or other metrics that give them a differential advantage over others, I think that’s something that…society might be sensitive to.”
Competition	“You don’t want to end up in a situation where you basically need to have DBS to succeed as a college student or something.”
Unequal access	It’s an access issue. If we had a universal health care system where everyone had equal access to those types of procedures, it would be a different story. – R_014

## Results

### Researchers See High Potential for Using Adaptive Deep Brain Stimulation for Enhancement

To the question of whether aDBS could be used for enhancement, a majority of researchers (70%) responded yes (Table 2). A few respondents expressed high confidence, such as one respondent who stated “I have no doubt that you could probably enhance people’s performance by modulating somehow the nervous system. I’m sure you can.” Some pointed out that the use of aDBS for enhancement is already happening. One respondent said, “We’ve already seen funding announcements for this sort of thing from DARPA, for example, for devices that are meant for healthy people to make them better.” Others suggested that the demand for enhancement using aDBS is likely to be prominent in certain arenas like sports and the military. One researcher argued, “If you did it in sports, I think the potential for it is far beyond that of performance enhancing drugs,” while another pointed out “It could also be used to create warriors that aren’t bothered by severe stress or catastrophic human loss…”. Others stated that aDBS could potentially be used for cognitive, mood or even moral enhancement, with one respondents saying, “It could actually enhance memory in patients without Alzheimer’s” and another stating “[There’s] memory, [and] you could imagine other kinds of cognitive enhancement, maybe motor enhancement [or] moral enhancement, like enhancing us to be better people.”

Some researchers believe that the use of aDBS for enhancement has already begun, as evidenced by certain ongoing aDBS studies. As one respondent said, “It was the DARPA initiative that is behind this, and I don’t think they’re doing this from the kindness of their heart, for wanting to help people with Parkinson’s, I think they’re doing this for military purposes.” Another commented on the existing power of aDBS for enhancing pleasure in ways that fall outside the scope of treatment:

*[Even now], we turn on a lead and the patient is smiling on one part of their face and getting happy and euphoric. And we say whoa, get a timeout*… *like we need to have an ethical rule as we move forward, an ethical override principle for this, as this is very powerful technology*… *it could turn into plastic surgery.*

#### Technical Constraints on Use of Adaptive Deep Brain Stimulation for Enhancement

Others (22%) felt there is little to no potential for using aDBS for enhancement due to significant logistical, technological and even biological constraints. One researcher said, “Honestly, I really don’t see a big future on the enhancement side. We may help you reach your biological potential, but I don’t think it’s going to make any improvements on that.” Researchers explained that “enhancements” are often imprecisely defined and thus difficult to localize and target, particularly in patients without a clinical disorder. One respondent commented, “I don’t know what enhancing an already functional person would look like.” Another said,

*It depends on whether you come at it from a disease model or non-disease model. It’s hard to think about helping one specific cognitive function. We know that even when you train people on something over and over, it doesn’t tend to generalize*… *so it’s hard to think about what you would stimulate that might re-regulate the whole network.*

Another researcher poignantly admitted, “I have a certain understanding of pathologic circuitry, [but] I don’t have a good understanding of normal circuitry.”

In addition to overcoming uncertainties about what parts of a non-diseased brain to target for enhancement, researchers cited another critical technological challenge, which is to individually tailor aDBS to ensure personalized benefits. One respondent explained:

*It’s really individual. [Whether] you place your electrode two millimeters north or south can make a huge difference, [and] each person probably*… *responds in a slightly different way. It’s almost always an N of one study, looking at this person and how specific symptoms respond to specific stimulation parameters exactly where their electrode is, which makes it much harder to generalize.*

### Attitudes Toward Use of Adaptive Deep Brain Stimulation for Enhancement

**FIGURE 1:**
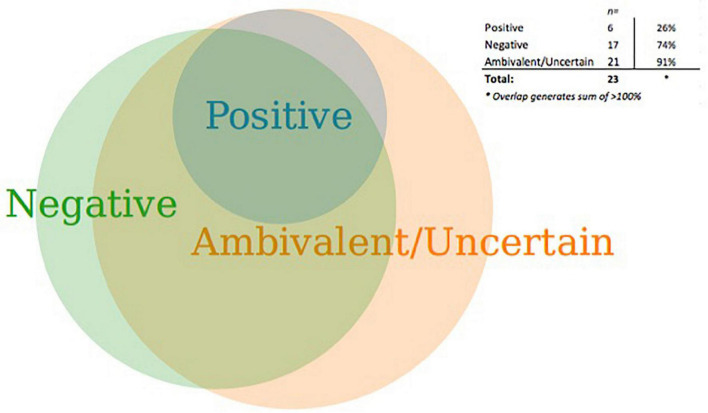
Attitudes toward use of adaptive deep brain stimulation for enhancement.

While a majority of aDBS researchers said they believe the use of aDBS for enhancement is imminent, nearly all (91%) also expressed ambivalence or uncertainty about using it for this purpose (Figure 1). We identified a respondent as having an “ambivalent/uncertain” attitude if he or she expressed comments that reflected both positive and negative appraisals and/or uncertainty. Note that the ambivalent/uncertain category overlaps with the positive and negative categories, with only a few non-overlapping responses. Even while some researchers held views that were in part positive (26%) or negative (74%), not a single respondent felt uniquely positive and only 1 respondent felt uniquely negative about it. Four respondents were uniquely uncertain and did not express attitudes that could be considered either positive or negative (e.g., “I am not sure.”).

### Primary Ethical Concerns About Using Adaptive Deep Brain Stimulation for Enhancement

#### Safety and Risk Considerations

Even if technical limitations to employing aDBS for enhancement could be overcome, most researchers (61%) said there would be significant safety concerns to address (Table 3). A primary safety consideration is the unavoidable risk associated with brain surgery. One researcher conveyed, “The risks of surgery are not insignificant. You could take someone who is essentially normal and you could really wreck them or kill them.” Others suggested the invasiveness of DBS may act as a deterrent for use in enhancement, with adaptive versions of DBS requiring even greater invasiveness than conventional DBS. One researcher emphasized that aDBS technology requires additional hardware (“a cortical strip electrode on the surface of the cortex in addition to their usual deep brain electrode”) that may lead patients to worry, “Is it going to increase my risk of infection… [or] of the device not working or breaking down?”

While current aDBS patients must weigh benefits against risks, this ratio may be particularly skewed for individuals who take on the risks of aDBS but do not suffer from disability or disorder. One respondent pointed out,

*The risk/benefit ratio in someone who has a diseased state, who is already becoming disabled by a pathological disorder, is very different than someone who is not disabled, is a normal, functioning human being. I would have significant ethical reservations and concerns about that.*

Another said, “Ethically I don’t think that’s okay. As a doctor, you wouldn’t want to do to surgery on someone that doesn’t have a problem.” Further, this risk benefit ratio is individualized and difficult to predict. One respondent claimed, “Everybody’s a little bit different, [and] predicting exactly how people are going to respond to DBS or adaptive DBS is tricky, because oftentimes we don’t know until we implant them and what their stimulation parameters are, as to how much benefit they’re going to get.”

#### Enhancement as Unnecessary, Unnatural or Aberrant

Many researchers (43%) felt that, even if these risks were minimized, they had other ethical reservations about using such a powerful technology for what many researchers characterized as non-essential aims. One researcher said, “If it’s to enhance treatment, of course, why not? But if it’s to be a better athlete or a better student, no, I don’t like that at all. I think that crosses ethical boundaries… [it] is a little ominous.” One researcher expressed the belief that allowing DBS for enhancement constitutes “an ethical gray area” with the potential to “really change the game of humanity.” Another highlighted something “unnatural” about the use of DBS for enhancement, commenting, “To me it’s a little bit like people having too many plastic surgeries. There’s something a little bit, I don’t know, grotesque about it.” The notion of changing fundamental aspects of a person’s character or sociability was also cited as off-putting, with one researcher saying, “I don’t think we want to change the whole make up [of] people who are just not having as much success or as much [*sic*] positive interactions, [and] they are seeking interventions to counteract that.” Another said, “I’m more okay with cognitive enhancements than changing who someone is.” Some researchers felt concerned that military applications in particular could disturb individual’s purported “free will” or change how human beings naturally respond to their environments, with one respondent expressing disapproval of any attempt to “create people [who], no matter what happens to them or what they’re asked to do, they don’t get traumatic harm from it,” later adding that, “Our conscience is important when we have people fighting, right? Otherwise we have atrocities.”

#### Fairness, Equality and Distributive Justice

Over a third of respondents (35%) raised concerns about potential socioeconomic consequences of allowing use of aDBS for enhancement, particularly unfair advantages conferred by the technology that could further exacerbate existing socioeconomic inequalities. One researcher commented, “It’s going to be very costly, and probably not coverable by insurance.” Another asked, “Should [individuals] who can afford it be able to do it, should it prove to really affect people in a way that provides them with unfair advantages societally?” Many researchers highlighted the high potential for such a scenario to “exacerbate class divisions.” One researcher said, “People of means could augment their abilities and their children’s abilities. The reproduction of social inequalities would be even more amplified than already exists in society. That’s problematic.” Other aDBS researchers raised concerns that, if enhancement using aDBS eventually became too widespread, it could potentially become a requirement for attaining competitive success. These researchers emphasized the ethical importance of fair access, with one researcher explaining: “Talk about enhancement really needs to revolve around access to enhancement. It doesn’t ethically matter if you’re able to enhance some attribute as long as everyone can enhance that attribute.”

A minority of researchers (26%) held partially positive views. Some offered an alternative perspective in emphasizing personal liberty, whereby individuals with the means to pursue aDBS for enhancement should not be barred from doing so. One respondent questioned, “If you’ve got the money for it, and you’re willing to take whatever risks that involves, then why not?” Another said, “I’m generally in favor of people being able to improve their lives with medical technology. I would see this as similar to taking stimulants, at least for what sort of enhancements you would have.” Other researchers felt less repelled than their colleagues by the idea of aDBS for enhancement, with some saying that there may be strong ethical justifications in certain circumstances (e.g., in military applications). One respondent said, “For our warriors? Heck, they’re putting their lives on the line they’re protecting their country. If I had something that I could put in their helmet that would make them more effective soldiers, I would support that.”

## Discussion

A glimpse at the unique insider perspectives of aDBS researchers reveals a collective sentiment that aDBS has high potential to be used for enhancement purposes. Many pointed to the fact that aDBS is already being developed by research entities (e.g., DARPA) for non-clinical purposes, including military applications and will soon be in demand for a portion of the consumer public undeterred by its invasive nature. Some echoed outlooks from scholars (Jasanoff, 2015) and industry (Neuralink Inc, 2022) that this minority may eventually grow to become a majority as acceptance of and optimism about the integration of technology in our daily lives continues to expand. However, many researchers said that developing this technology for enhancement is not and should not be endorsed by research and clinical communities, citing surgical risks and an imbalanced risk-benefit ratio when offering aDBS to healthy or “normally” functioning individuals with potentially less to gain and more to lose compared to patients with a treatment-resistant disorder.

A small number of respondents expressed aversion toward fundamentally altering the nature of personhood, a perspective in line with the view memorably espoused in the 2003 report by the President’s Council of Bioethics (Kass, 2003). However, this perspective was not widespread. Further, while researchers in our sample did express concerns about potential impacts on fairness, equality, and distributive justice that have been raised extensively in the larger literature on enhancement (Goering and Yuste, 2016; Wasserman and Aas, 2016; Hopkins and Fiser, 2017), these concerns did not appear to be central ethical preoccupations.

Instead, researchers and clinicians appeared to focus most on issues of safety and efficacy in their evaluations of whether aDBS could ethically be used for enhancement. Given the current state of the science, respondents say they do not yet have a good understanding of how to stimulate a healthy brain in ways that could safely and effectively evoke a generalized enhancement capacity such as “improved memory” (let alone more controversial and difficult-to-define concepts such as “cognitive functioning” or “intelligence”). They suggest that better definitions of what constitutes a given enhancement are necessary precursors to applying aDBS for enhancement. However, even equipped with more precise conceptualizations, researchers feel they still have an insufficient understanding of which areas of the brain are implicated in certain tasks or capacities. While discoveries in brain mapping have gained significant traction with the innovation of diffusion (Basser and Jones, 2002) and functional (Van Dijk et al., 2010) MRI technologies (particularly in association with the Human Connectome Project) (Van Essen et al., 2013) and continue to advance through novel chemo- and optogenetic methodologies (Galvan et al., 2018), our respondents said they feel that a greater understanding of brain functioning and localization is needed before aDBS technologies can be safely and effectively used for enhancement. The capacity to evoke a range of desired enhancements requires a firmer understanding of where specific capacities are localized (or distributed) in the brain. Their concerns echo limitations of DBS that have been outlined in the clinical literature (Lozano et al., 2019), including the need not only to improve targeting but also to avoid stimulation of the brain in unwanted ways, requiring a high degree of spatial and temporal resolution that is not feasible using current technology. Compared to hardware and software used for spinal cord stimulation in pain management, for example, which benefits from new waveforms and software strategies such as high-frequency, high-density and burst stimulation, aDBS technology remains a generation behind (Lozano et al., 2019). While advancements are being made in thin-film technology and the development of multi-contact, flexible and more biocompatible electrodes to provide better control of the stimulation field and high-resolution readouts of neural circuit function, these have not yet been implemented in aDBS systems (Lozano et al., 2019).

The current lack of resolution raises both practical and ethical concerns. Some neurotechnology researchers such as Wurzman et al. (2016) have cautioned that “stimulation affects more of the brain than a user may think… [and] extends well beyond the regions beneath the electrodes.” Indeed, research demonstrates that while aDBS focuses on anatomic targets typically on the order of millimeters, it can have profound influences on brain-wide networks (Ballanger et al., 2009; Laxton et al., 2010; Lipsman et al., 2013), sometimes entailing significant, negative side effects (Tripoliti et al., 2011; Huebl et al., 2015). Our respondents agreed almost unanimously that aDBS ideally should not realistically be considered for enhancement until researchers achieve a better understanding of brain localization and functioning, of long-term effects on targeted and secondarily affected neural networks, and of how to further improve stimulation resolution.

### How Are Researchers’ Ethical Concerns Unique?

While the set of concerns expressed by our researcher respondents overlap with those outlined in existing discourses on neuro-enhancements, we found notable differences. One is that, while our respondents raised numerous technical and logistic challenges to the effective (and safe) use of aDBS for enhancement, they did not focus on some of the more inherent features of aDBS that might make its use for enhancement controversial. For example, issues of data privacy and security of brain activity data that have been raised (Ienca et al., 2018) as substantial concerns with next-generation neuromodulation devices like aDBS were not mentioned by the researchers in our sample in response to questions about enhancement [though they did discuss these issues in response to other questions about broader ethical considerations of aDBS, which we report on elsewhere (Muñoz et al., 2020)]. Likewise, concerns about the capacity of aDBS to monitor brain activity as an essential part of its closed-loop system were not raised in relation to enhancement specifically. This is somewhat surprising, given the extent of commercial interest in determining how to monitor, monetize, and commodify consumer brain activity data (Dasgupta, 2020) on the one hand, and calls to heavily regulate or even ban monetization of such data, on the other (Yuste et al., 2017). While currently lacking in interpretability, brain activity data may soon provide clues into the dispositional or behavioral characteristics of aDBS users that could in turn be used to generate profit, assuming these data could be sufficiently accompanied by the contextual information critical to understanding their meaning (Wexler, 2019). Hypothetically speaking, brain activity data could be sold or licensed by a device company or related affiliates, or consumers could one day choose to sell or license their own data for profit [assuming that they would have access to data from their own brains, which is not a given, and assuming such sales are not made unlawful through legislation (Wexler, 2019)].

That our research respondents did not spontaneously cite these as salient concerns is telling. It is possible that that their main concerns, which focused more on safety, norms and social consequences, reflect their social location within a knowledge-seeking and -generating space – the academic health science center – rather than a profit-seeking, commercial enterprise. It is also likely that researchers’ expert knowledge about the current state of the field and its limitations highlight feasibility and safety as paramount concerns, and render comparatively less salient the oft-cited concerns about brain data leaks (Ienca et al., 2018), exploitation, and the potential perils of mind-reading (Wexler and Thibault, 2019; Zuk and Lázaro-Muñoz, 2019b). Even less worrying to researchers is the possibility that commercially available aDBS systems might eventually open up the ability to merge our minds with artificially intelligent devices, despite the excitement and frenzy this idea has generated among scholars, scientists and engineers (Fourneret, 2020). Researchers working within the field of aDBS appear more immediately concerned with efficacy and safety over hypothetical scenarios. This focus on the proximate versus the distant or ultimate invokes a plea that Walsh (2013) made to his fellow neuroscientists nearly a decade ago to avoid “hyping” their results and to conscientiously distinguish “between what is scientifically interesting [versus] clinically or recreationally possible” (Walsh, 2013).

Some have argued that scholars and ethicists are “jumping the gun” with respect to worrying about the potential negative impacts of aDBS and other emerging neurotechnologies. Comments from our researcher respondents seem to corroborate this view, insofar as they believe not all concerns raised in the literature in relation to enhancement are salient in the near-term. But just how far are we from being able to overcome the challenges that aDBS researchers cite as current limitations? A serious response to this question is beyond the scope of this paper; but the short answer seems to be neither “right around the corner” nor “beyond our lifetime.” Neuroscientists are discovering greater complexity in brain connectivity and functioning that challenge the simplistic notion that aDBS can stimulate a region of the brain that governs a cognitive capacity or task (e.g., mathematical reasoning) to elicit a desired phenotypic response. In reality, we are discovering that the brain is characterized by far more functional heterogeneity and multiplicity of brain areas, currently being explored by new “gradient” or “connectopic mapping” approaches that counter previous assumptions that the brain may be parceled into patches of “piece-wise constant connectivity” (Haak and Beckmann, 2020).

While the brain’s complexity poses current limitations on the extent to which aDBS can be effectively used for enhancement, there are other reasons to believe the prospect might not be too far off. The precise localization of brain activity and functioning may not be necessary for eliciting certain enhancements. Evidence from the use of transcranial stimulation devices among athletes suggests that delivering stimulation while performing a task helps the brain to build new connections as it learns a skill. One study showed that Nordic ski jumpers who received transcranial direct stimulation (tDCS) while practicing jumping onto an unstable platform improved the athletes’ jumping force by 70% and their coordination by 80%, compared with the sham group (Reardon, 2016). Results from a similar study suggest that delivering over-the-scalp stimulation while performing a movement task can reduce an athlete’s ability to perceive muscular fatigue (Cogiamanian et al., 2007). These findings suggest that stimulation may not have to be precisely targeted or delivered with high resolution for all types of enhancement. Early applications of aDBS for enhancement may target a range of enhancement capacities that are easier to actuate compared to other types of enhancement (e.g., memory retrieval or mood modification) that may require more sophisticated understandings of brain function. While we may be far from using aDBS for some types of enhancement, for others, such as modulation of mood, aDBS has already been shown to be possible (Synofzik et al., 2012). Indeed, psychosurgical effects on mood are well documented in the historical record since the 1940s (Freeman, 1948). With regards to aDBS, the impacts on mood once observed to be accidental side effects among certain patients [e.g., with Parkinson’s (Funkiewiez et al., 2003)] are now controlled main effects for the treatment of certain psychiatry disorders, including refractory major depression (Schlaepfer et al., 2008).

How close we are to being able to use aDBS for enhancement may also be influenced by improvements in hardware on the horizon. The current state-of-the-art DBS lead, consisting of a linear stack of four or more cylindrical electrode contacts, will eventually be replaced with multi-contact DBS electrodes and thin-film probe technology, opening the door to delivering stimulation with significantly higher density and resolution (Lozano et al., 2019). In parallel but related fields, basic research is also underway to explore alternatives to electric stimulation using opto- and chemogenetics to further improve the spatial and temporal resolution of neural stimulation (Montagni et al., 2019).

## Limitations

Our findings may not be generalizable to all researchers and researcher clinicians operating in the field. Although we ensured recruitment of researchers who have various professional roles in aDBS trials, 78% of the sample identified as white, reflecting a lack of racial and ethnic representation in our sample. We cannot be certain that selection bias (who was invited and agreed to participate) did not play a role in response patterns. Further, we identified concerns based on researchers’ responses to two direct questions about enhancement as well as their spontaneous mention of enhancement within a larger interview guide focused on exploring ethical issues in use of aDBS in research trials (Muñoz et al., 2020). We cannot be sure they represent a full range of aDBS researchers’ expressed and unexpressed concerns. The salience and representativeness of their expressed concerns across a wider population of aDBS researchers could be confirmed by more structured survey methods. Finally, researchers’ perspectives reflect their specific expertise and should be understood within a broader context of stakeholder perspectives, which may offer wider insights into social and cultural impacts of using aDBS for enhancement.

## Conclusion

Insights from aDBS researchers reveal that feasible application of aDBS for enhancement remains far off but is not out of reach, with a vast majority (70%) seeing clear potential. These experts highlight what they characterize as fundamental challenges and concerns related to feasibility, efficacy and consumer safety. While acknowledging certain controversies highlighted by many scholars and ethicists with respect to potential impacts of using aDBS for enhancement on personhood and society, aDBS researchers remind us that near-term research and policy should focus on concerns that carry the most serious and most probable risks. A principal hazard of attending to concerns that may be far-off or less immediately worrisome is that it potentially distracts us from engaging with more pressing and proximate risks of using aDBS for enhancement.

## Data Availability Statement

The raw data supporting the conclusions of this article will be made available by the authors, without undue reservation.

## Ethics Statement

The studies involving human participants were reviewed and approved by the Institutional Review Board for Baylor College of Medicine. The patients/participants provided their written informed consent to participate in this study.

## Author Contributions

KK-Q, PZ, GL-M, LT, KM, SP, AM, and DS-M contributed to the writing of this manuscript. KK-Q and LK contributed to data analysis. LT, SO, CS, RH, KM, and JR contributed to data collection and data management. All authors are in agreement with the contents of this manuscript.

## Conflict of Interest

The authors declare that the research was conducted in the absence of any commercial or financial relationships that could be construed as a potential conflict of interest.

## Publisher’s Note

All claims expressed in this article are solely those of the authors and do not necessarily represent those of their affiliated organizations, or those of the publisher, the editors and the reviewers. Any product that may be evaluated in this article, or claim that may be made by its manufacturer, is not guaranteed or endorsed by the publisher.
